# Endometrial injury, the quality of embryos, and blastocyst transfer are the most important prognostic factors for in vitro fertilization success after previous repeated unsuccessful attempts

**DOI:** 10.1007/s10815-017-0916-4

**Published:** 2017-04-06

**Authors:** Milan Reljič, Jure Knez, Vilma Kovač, Borut Kovačič

**Affiliations:** Department of Reproductive Medicine and Gynecologic Endocrinology, Clinic for Gynecology and Perinatology, University Medical Centre Maribor, Ljubljanska 5, 2000 Maribor, Slovenia

**Keywords:** Recurrent IVF failure, Local endometrial injury, Blastocyst transfer

## Abstract

**Purpose:**

The purpose of this study was to find out the most important prognostic factors for achieving a pregnancy after in vitro fertilization (IVF) in women with history of repeated unsuccessful IVF attempts.

**Methods:**

We analyzed factors affecting pregnancy rate in a retrospective study including 429 IVF/ICSI cycles performed in women younger than 40 years with at least three previous consecutive failed IVF/ICSI attempts.

**Results:**

Clinical pregnancy was observed in 140/429 (32.6%) cycles. Clinical pregnancy rate (CPR) was significantly higher in cycles with LEI compared to cycles without LEI before embryo transfer (44.4 vs 26.54%, *p* = 0.007). The CPR was also higher in cycles with day 5 blastocyst- compared with day 3 cleavage-stage embryo transfers (45.51 vs 26.54%, *p* < 0.001). In multivariate logistic regression model, only transfer of at least one good quality embryo (OR = 4.32, 95% CI 2.41–7.73), local endometrial injury (OR = 1.73, 95% CI 1.02–2.92), and transfer on day 5 (OR = 3.02, 95% CI 1.53–5.94) remained important independent prognostic factors for clinical pregnancy.

**Conclusions:**

These results suggest that hysteroscopy with local injury to the endometrium prior to ovarian stimulation for IVF/ICSI can improve implantation and pregnancy rates in women experiencing recurrent IVF failure. However, large studies are needed to confirm these findings.

## Introduction

Repeated IVF treatment failure is very frustrating to the patients as well as to the clinicians. Even in subsequent IVF/ICSI cycles, lower pregnancy rates can be expected in these patients [1, 2]. Many research efforts have been invested to find the factors that affect the pregnancy rate in these couples, not only to advise patients about treatment success but also to propose methods of treatment that would improve their possibility of conception. The woman’s age, the indication for IVF, ovarian reserve, the treatment protocol employed, uterine pathology, immunological factors, number of embryos transferred, number of available embryos, embryo quality, embryo transfer technique, sperm quality, and luteal phase support were identified as interfering with successful implantation and contributing to recurrent failure. However, reduced endometrium receptivity and low embryo quality are thought to be the most important factors [3].

Several approaches have been proposed to improve success rates in these women, including blastocyst culture, local endometrial injury (LEI), assisted hatching, sequential transfer, co-culture system, zygote intrafallopian transfer, pre-implantation genetic screening (PGS), intracytoplasmic morphologically selected sperm injection (IMSI) for treating male infertility, etc. However, the benefit of these methods has not been confirmed in properly designed studies. LEI and blastocyst transfer are the methods with most evidence in the literature.

A LEI in the luteal phase of the cycle preceding ovarian stimulation for IVF has been associated with improved implantation in women with unexplained repeated implantation failure (RIF). Systematic review of the literature and meta-analysis of seven studies published until 2012 showed that pregnancy rate in women who have undergone LEI was 71% higher compared to pregnancy rate in women with no intervention. (RR 1.71, 95% CI 1.44–2.02) [4]. In a subsequent Cochrane review which included 14 studies, LEI was also associated with higher pregnancy rate, but the benefit of this procedure was less evident (RR 1.34, 95% CI 1.21–1.61) and the authors concluded that more evidence from well-designed trials is needed [5]. The possible bias is also that there is no uniform definition of RIF. Not all follow the recommendation that RIF is not the same as repeated IVF treatment failure and that the definition of RIF requires good-quality embryos to be transferred [6, 7]. The effect of LEI still remains controversial and the subgroup of patients with RIF who would mostly benefit from LEI still needs to be identified. Despite these concerns it was found in recent survey that in women with RIF, 92% of clinicians would recommend LEI [8].

In patients with repeated IVF treatment failure, blastocyst transfer is also often advised. This recommendation are based on studies that have found higher implantation rate for women who underwent blastocyst transfer compared to those in whom embryos were transferred on day 2 or day 3 [9–12]. But in some others, no beneficial effect of blastocyst transfer was reported [2, 13].

Despite that, there is some evidence to support both mentioned approaches; LEI and blastocyst transfer together with other prognostic factors affecting pregnancy rate have not yet been studied using multiple logistic regression model in unselected group of women with repeated IVF failure.

## Materials and methods

We included 429 IVF/ICSI cycles performed in women younger than 40 years with at least three previous consecutive failed IVF/ICSI attempts in a retrospective study. The data were retrieved from the database of all IVF/ICSI cycles conducted at the Department for Reproductive Medicine, University Medical Centre, Maribor, Slovenia, from January 2014 to December 2015. Cycles in women with uterine pathology, with intrauterine procedures in the last 3 months, with poor ovarian response according to the Bologna criteria, and those without embryo transfer were excluded. According to the doctor-patient agreement some women were scheduled for LEI. In all women, LEI was performed in the luteal phase of the cycle preceding ovarian stimulation in an office hysteroscopy setting. Multiple endometrial injuries approximately 2 mm in depth and width in the upper part of the uterine cavity were performed using grasping forceps or scissors.

Patients underwent ovarian stimulation using protocols with combination of GnRH agonist/GnRH antagonist and recombinant FSH (Gonal-F, Serono International SA, Geneva, Switzerland)/HMG (Menopur, Ferring Pharmaceuticals Inc., Saint-Prex, Switzerland) that were previously described in detail [14]. After oocyte fertilization using IVF or ICSI procedure, embryos were cultured in the BlastAssist extended culture media (Origio, Denmark). Embryo quality was assessed by an experienced embryologist at day 2 and 3 after oocyte fertilization. Day 5 blastocyst transfer was performed if more than three optimal embryos were available on day 3 according to our standard policies. After consultation with the patients, time of embryo transfer was adjusted to day 3 or day 5 according to doctor-patient agreement. Blastocysts were graded according to our established grading system 5 days after oocyte fertilization [15, 16]. In brief, the blastocyst was considered optimal if it was fully expanded and the blastocoel completely filled the embryo. It contained a cohesive trophectoderm and a compact inner cell mass (ICM). No more than three embryos on day 3 and no more than two embryos on day 5 were transferred. Surplus blastocysts, not selected for transfer were cryopreserved. After embryo transfer, patients received luteal-phase support with 600 mg of vaginal progesterone daily (Utrogestan, Ferring Pharmaceuticals Inc., Saint-Prex, Switzerland). Serum hCG level was measured 16 days after oocyte pick-up and ultrasound was performed 2 weeks later if the blood test confirmed the pregnancy. Clinical pregnancy was defined as the presence of a gestational sac with a fetal heartbeat.

Patients’ and cycles’ characteristics were compared between the conception and non-conception cycles. Statistical analysis was performed with Statistica 8.0 data software system analysis (Stat Soft Inc., Tulsa, OK, USA). The normal distribution of numeric variables was determined by the Shapiro-Wilk test. Student’s *t* test or the Mann-Whitney *U* test was used to assess these variables, depending on the data distribution. Mean and standard deviation for each continuous variable were calculated. Cross-tables and chi-square analysis were employed in the evaluation of the categorical data. The association between patients’/cycles’ characteristics and clinical pregnancy were also analyzed with univariate logistic regression. Variables proven statistically significant by univariate logistic analysis were tested with multiple logistic regression model. Odds ratios and their 95% confidence intervals (CIs) were calculated. *p* value < 0.05 was considered statistically significant.

## Results

Among 429 included cycles, clinical pregnancy was observed in 140 (32.6%) of them. LEI were performed in 90 (23.3%) cycles preceding embryo transfer. The clinical pregnancy rate (CPR) in this subgroup was significantly higher compared with CPR in cycles without LEI (44.4 vs 26.54%, *p* = 0.007). CPR was higher after LEI in cycles with day 5 embryo transfer (54.8 vs 37.12%) as well as in cycles with day 3 embryo transfer (35.4 vs 25.7%). Embryo transfer on day 5 was done in 155 (36.1%) cycles and resulted in 71 clinical pregnancies. The CPR was higher in cycles with day 5 compared with CPR in cycles with day 3 embryo transfer (45.51 vs 26.54%, *p* < 0.001). Embryo transfer of at least one good quality embryo was performed in 271 (63.17%) cycles achieving significantly higher CPR than after transfer of lower quality embryos (39.48 vs 20.88%, *p* < 0.001). There were no statistically significant differences between conception and non-conception cycles in women’s age, number of previous IVF cycles, causes of infertility and number of transferred embryos. Statistically significant different total dose of gonadotrophins, number of oocytes retrieved, number of embryos, number of good quality embryos on day two, number of blastocysts, number of freezing blastocysts, proportion of cycles with embryo freezing, proportion of cycles with embryo transfer of at least one good quality embryo, proportion of cycles with day five ET and proportion of cycles with LEI were observed in conception compared to non-conception cycles (Table 1).

**Table 1:** Patients’ and cycles’ characteristics

	Conception IVF cycles *N* = 140	Non-conception IVF cycles *N* = 289	*p* value
Age (years)	33.82 ± 3.41	34.52 ± 3.07	NS
No. of previous cycles	4.30 ± 1.44	4.75 ± 2.23	NS
Male factor infertility (*N*, %)	68 (48.57)	138 (47.75)	NS
Total gonadotrophin dose (X 75 IU)	28.82 ± 10.21	31.21 ± 11.68	0.02
No. of oocytes retrieved	11.61 ± 5.19	9.93 ± 5.79	<0.001
ICSI (*N*, %)	93 (66.42)	200 (69.20)	NS
No. of embryos on day 2	6.95 ± 3.78	5.34 ± 3.52	<0.001
No. of good-quality embryos (day2)	4.63 ± 3.63	3.35 ± 3.12	<0.001
No. of blastocysts	2.76 ± 2.90	1.47 ± 2.46	<0.001
Day 5 embryo transfer (%)	71 (50.71)	84 (30.43)	<0.001
Number of embryos transferred	1.89 ± 0.49	1.79 ± 0.65	NS
ET of at least one quality embryo (*N*, %)	107 (76.43)	164 (56.75)	<0,001
Cycles with embryo cryopreservation (*N*, %)	69 (49.29)	195 (32.53)	<0.001
Number of embryos cryopreserved	1.46 ± 2.13	0,80 ± 1.71	<0.001
Local endometrial injury (*N*, %)	40 (28.57)	50 (17.30)	0.01

*ICSI* intracytoplasmic sperm injection, *ET* embryo transfer, *NS* not significant

These parameters were also found to be associated with clinical pregnancy using univariate logistic regression. In multivariate logistic regression model, only transfer of at least one good-quality embryo, local endometrial injury and transfer on day 5 remained important independent prognostic factors for clinical pregnancy (Table 2).

**Table 2:** Univariate and multivariate logistic regression analysis assessing predictors of clinical pregnancy after in-vitro-fertilization (IVF) in women with history of at least three consecutive unsuccessful IVF attempts (coefficient = −2.31, final loss: 237.45, chi^2^ (10) = 63.16, *p* < 0.001)

Independent variable	Univariate regression		Multivariate regression	
	Odds ratio (95% CI)	*p* value	Odds ratio (95% CI)	*p* value
Total gonadotrophin dose	0.26 (0.06–0.94)	0.04	0.99 (0.97–1.01)	0.31
Number of oocytes retrieved	4.67 (1.61–13.50)	0.004	0.98 (0.93–1.04)	0.61
Number of embryos on day 2	14.68 (4.06–53.05)	>0.001	1.12 (0.99–1.28)	0.07
Number of good-quality embryos (day2)	12.67 (3.44–46.57)	>0.001	0.95 (0.83–1.09)	0.46
Number of blastocysts	16.46 (4.81–56.32)	>0.001	1.09 (0.90–1.33)	0.35
ET of at least one good quality embryo	2.47 (1.56–3.89)	>0.001	4.32 (2.41–7.73)	<0.001
Cycles with embryo cryopreservation	2.02 (1.33–3.05)	>0.001	1.05 (0.55–1.99)	0.89
Number of cryopreserved embryos	15.11(2.93–77.71)	0.001	0.84 (0.65–1.09)	0.19
Local endometrial injury	1.91 (1.18–3.08)	0.008	1.73 (1.02–2.92)	0.040
Day 5 embryo transfer	2.51 (1.65–3.81)	>0.001	3.02 (1.53–5.94)	0.001

*ET* embryo transfer, *CI* confidence interval

We have also performed the multivariate regression analysis including only the two significant variables at the same time. By using this approach, these three variables remained independent prognostic factors for clinical pregnancy in each of the models.

## Discussion

We have shown that local endometrial injury and blastocyst transfer are important independent prognostic factors for achieving a clinical pregnancy after previous recurrent failed IVF. In our study, we have conducted a multivariable analysis in order to account for all important factors that could affect the pregnancy rate together in the current cycle. Poor ovarian response according to the Bologna criteria and advanced age are very well-documented and common causes of recurrent IVF failure, and these cycles were excluded from study. We only included cycles with embryo transfer, so that a relatively high clinical pregnancy rate of 32.6% is not unexpected.

In conception cycles compared to non-conception cycles, higher number of oocytes retrieved, all available embryos, good-quality embryos, blastocysts, freezing surplus blastocysts, proportion of cycles with embryo transfer of at least one good-quality embryo, and lower dose of gonadotrophins were all related to a higher clinical pregnancy rate which is in consistence with findings of other authors [3, 15–18]. Women who did not get pregnant were older that those who did, but this difference did not reach statistical significance. The main reason could be the fact that the pregnancy rate after IVF decreased with increasing age, but decline is most pronounced after 40 years, and this group of women was excluded from our study. According to our findings, the number of transferred embryos is not an important factor affecting pregnancy rate. In most cycles, two embryos were transferred, and as it was demonstrated in a recent systematic review, transferring more than two embryos is not associated with a higher birth rate [19].

Blastocyst transfer was an independent prognostic factor for clinical pregnancy in our study (OR = 3.02 (1.53–5.94)). The possible benefit of blastocyst transfer is better embryo selection and synchronization of embryo stage with the endometrium [20]. In our study, we calculated pregnancy rate per transfer instead of per cycle. Considering this aspect, better embryo selection may be an even more important aspect of blastocyst transfer in our group of patients. Another reason for this assumption is also our transfer policy, which means that day 5 transfer is performed only if more than three optimal embryos were available on day 3. Nonetheless, multiple logistic regression model demonstrated that day 5 is an independent prognostic factor for pregnancy, irrespectively, to the number and quality of embryos on day 2. However, from the results of this study, it is impossible to assert that the same embryo has a better chance to implant if it is transferred on day 5 instead of day 3. In our previous study where pregnancy rate per cycle was compared between day 2 and day 5 transfer in cycles with one or two developed embryos, no differences were found [21, 22], but those studies included unselected group of women and not only patients with repeated IVF treatment failure.

Several studies have shown that local endometrial injury (LEI) can improve implantation rates in patients with unexplained repeated implantation failure. Still, there is a lot of debate on this subject due to heterogeneity in the design of the studies. Despite the lack of uniformity of the definition, relatively recent suggested consensus which is most commonly applied today is that RIF should be defined as a failure of implantation in at least three consecutive IVF attempts in which 1–2 embryos of high-grade quality are transferred in each cycle [6, 23]. Our study confirmed the positive effect of LEI in patients with recurrent IVF failure, since pregnancy rate in women who have undergone hysteroscopy and LEI was 73% higher compared to pregnancy rate in women with no intervention (OR 1.73, 95% CI 1.02–2.92). The local injury to the endometrium can be induced by endometrial biopsy (scratch) or hysteroscopy, and it has been shown that endometrial biopsy is twice as effective as opposed to hysteroscopy [4]. In our study, women underwent hysteroscopy not only to perform endometrial biopsy under visual control, but also to exclude pathology of uterine cavity, which can also contribute to improving the chances of conception. LEI was performed according to doctor-patient agreement, meaning that not every patient with recurrent IVF failure underwent this procedure and LEI was also done in patients who do not completely fulfill the recommended criteria for RIF [6, 23], regarding high-grade embryo quality.

A limitation of the study is that LEI was performed in a relatively low proportion of patients with previous unsuccessful IVF attempts. Due to the retrospective nature of the study and non-specific criteria for LEI, there is a possibility of a selection bias. Nonetheless, we used a multiple regression model in order to account for possible confounders and to overcome this methodological problem. Limitation of our study is also that not all factors that could interfere with implantation were included in analysis.

Our results suggest that quality of transferred embryos is the most important prognostic factor for conception and that blastocyst transfer and LEI should be recommended to patients with repeated IVF failure in order to improve the pregnancy rate. Larger prospective multi-center studies are needed to confirm these findings.
